# Implementation of a medical genomics program for rare diseases in Uruguay

**DOI:** 10.1186/s13023-026-04387-2

**Published:** 2026-06-24

**Authors:** Camila Simoes, María Fernanda Domínguez, Soledad Rodriguez, Mariana Moraes, María Paula Frade, Mariana Brandes, Martín Graña, Lucía Sosa, Santiago Fontenla, Alejandra Tapié, Nicolás Dell’Oca, Olaf Riess, José Tort, Hugo Naya, Víctor Raggio, Lucia Spangenberg

**Affiliations:** 1 Bioinformatics Unit, Institut Pasteur de Montevideo, Montevideo, Uruguay; 2 Departamento Básico de Medicina, Hospital de Clínicas, Facultad de Medicina, Universidad de la República, Montevideo, Uruguay; 3 Departamento de Genética, Facultad de Medicina, Universidad de la República, Montevideo, Uruguay; 4 Institute of Medical Genetics and Applied Genomics, University of Tübingen, Tübingen, Germany; 5 Departamento de Producción Animal y Pasturas, Facultad de Agronomía, Universidad de la República, Montevideo, Uruguay

**Keywords:** Genetic diagnostic, Exome sequencing, Genome sequencing, Rare diseases, Medical genomics

## Abstract

**Background:**

Rare diseases (RDs) affect an estimated 7% of the global population and comprise ~7,000 heterogeneous conditions, most of which have a genetic etiology. Despite their collective burden, RDs pose major diagnostic challenges, often resulting in prolonged diagnostic odysseys with substantial clinical, emotional, and economic consequences. Next generation sequencing technologies such as whole exome (WES) and whole genome sequencing (WGS) have transformed RD diagnostics in high-resource settings. In contrast, the use of such technologies in Latin America remains uneven due to structural, regulatory, and economic constraints. In addition, the underrepresentation of admixed populations in genomic databases further complicates variant interpretation, reducing the diagnostic yield. In this context, we created the first genomic medicine program for rare diseases in Uruguay to address these challenges by implementing next-generation sequencing (NGS) strategies for diagnosis.

**Material and methods:**

Whole exome sequencing (WES) and whole genome sequencing (WGS) were performed on a cohort of 203 patients with suspected RD belonging to the Uruguayan public healthcare system. Variant prioritization was conducted using established bioinformatics pipelines integrating population frequency filtering, inheritance models, functional impact, and phenotype-driven clinical interpretation. Implementation of population-aware variant interpretation strategies was critical in this admixed population, helping to reduce false-positive findings arising from the underrepresentation of Latin American populations in global reference databases. This framework was built through sustained collaboration between academic institutions and healthcare providers, with a strong emphasis on local capacity building in clinical genetics, molecular diagnostics, and bioinformatics.

**Results:**

To date, we have analyzed a total of 203 patients: 172 using WES, 29 using WGS, and 11 using mitochondrial DNA sequencing (9 of whom were also analyzed by WES). The overall diagnostic yield was 48% for WES and 31% for WGS. The program significantly shortened diagnostic trajectories and enabled actionable clinical insights in a substantial proportion of cases.

**Conclusions:**

Our experience shows that comprehensive genomic diagnostics for RDs can be successfully carried out in emerging genomic medicine settings through integrated workflows and sustained investment in human capital. This initiative represents a scalable model for other low income countries seeking to incorporate genomic medicine into public healthcare systems and highlights the importance of regional genomic data to improve diagnostic accuracy and equity.

**Supplementary Information:**

The online version contains supplementary material available at https://doi.org/10.1186/s13023-026-04387-2.

## Introduction

It is estimated that 7% of the population suffers from rare diseases (RDs), a heterogeneous group comprising around 7,000 different conditions, most of them of genetic origin [1–3]. While each condition individually is very uncommon (1 in 2,000), when considered together, they affect a large number of individuals [3–5]. At the regional level Latin America lacks a consensus definition of RD [6], in contrast to regions such as the European Union, where a unified prevalence threshold (<1 in 2,000 individuals) is widely adopted [3]. Regulatory frameworks differ substantially between countries, with some adopting the EU definition (e.g., Argentina, Chile, Mexico, Panama, and Uruguay), others applying alternative thresholds (as in Brazil or Colombia), and several countries without an official definition at all [6–9]. This heterogeneity hampers regional estimates of prevalence, the development of harmonized policies, and the creation of coordinated programs or registries.

Despite geographical differences and definitions, RDs consistently share several key features. Most of them affect children, have a significant impact on quality of life and life expectancy, are generally chronic, severe, and progressive, and most of them do not have a specific treatment. Additionally, due to their rare nature, they are generally a diagnostic challenge [4, 5, 10]. These challenges often result in a “diagnostic odyssey”, a prolonged process during which patients undergo repeated consultations with specialists and studies (including invasive procedures) and may remain without a definitive diagnosis. Several factors contribute to this trajectory, including the clinical heterogeneity of RDs, the nonspecific nature of early symptoms in many of them, and the limited exposure that most healthcare professionals have to these conditions [3, 4]. Because RDs affect very small and geographically dispersed patient populations, healthcare professionals often lack the familiarity, training, and time required to recognize them early in the clinical course [11, 12]. As a consequence, individuals with RD often face years of uncertainty, misdiagnoses, and substantial emotional, financial, and medical burdens that affect not only patients themselves, but also their families, clinicians, and the healthcare system [13, 14].

These diagnostic challenges and the fact that most RDs have a genetic etiology led to the widespread use of genomic technologies to more rapidly and accurately identify the underlying genetic causes. In particular whole exome sequencing (WES) has transformed both gene discovery and clinical diagnostics [15]. By targeting the 1%-2% of the genome it provides an efficient, scalable, and cost-effective approach for patients with suspected genetic disorders. Diagnostic success rates range from 25 to 58%, depending on the medical specialty in which they are applied [16]. In addition to WES, whole genome sequencing (WGS) offers a broader strategy by enabling the detection of genomic variation beyond coding regions [17], including structural variants, copy-number changes, repeat expansions, and noncoding regulatory variants [12, 18–20]. Recent studies have shown an increased diagnostic rate using short read WGS of ≈10% [21–23]. Another way to improve diagnostic efficiency is trio testing, i.e., testing both parents and the patient together, at a higher cost but allowing to detect inheritance patterns. Additionally, long-read WGS (LR-WGS) is emerging as another layer of resolution, contributing an extra 7–17% diagnostic yield in cases that remain unsolved after short-read WGS [21].

Early implementation of WES and WGS has consistently been shown to be cost-effective, reducing unnecessary investigations, shortening the diagnostic trajectory, and supporting timely clinical decision-making [24–27].

Recently, several Latin American groups have reported the outcomes of several years of efforts to incorporate genomic technologies into RD diagnostics, each adopting strategies shaped by local constraints and opportunities [14, 28–36]. Brazil progressively incorporated genomic diagnostics through exome-based clinical studies and the national Rare Genomes Project. Exome sequencing of 500 patients achieved a 31.6% diagnostic yield with significant clinical impact [35], while the Rare Genomes Project validated high-quality WGS for use within the public health system and applied it to > 1200 real-world cases [30]. Peru and Mexico have implemented clinical WGS through sustained academic-industry collaborations such as Illumina iHope Program, achieving diagnostic yields of 54–68% and demonstrating long-term clinical impact in resource-limited settings. Notably, Peru reported a 54.3% diagnostic rate in a national cohort [28], while Mexico achieved 68.3% in a first-tier WGS pilot for undiagnosed children [36]. Colombia, through a national multi-institutional effort, conducted the first Latin American study to systematically apply clinical WGS, analyzing 501 patients across 22 healthcare sites, with a 28.3% diagnostic yield, demonstrating added value over prior exome testing [14]. In Chile, the DECIPHERD program stands out as a structured, two-phase initiative that combines international training and subsequent local development. It adopted a hybrid model in which sequencing was outsourced while clinical and bioinformatic analyses were performed in-house, achieving a diagnostic yield of ~46% and demonstrating the feasibility of implementing exome-based diagnostics in settings with limited genomic infrastructure [34]. Together, these initiatives illustrate both the diversity of implementation strategies and the shared regional challenges: limited reimbursement mechanisms, restricted sequencing capacity, unequal access to specialists, and the need for long-term integration of genomic diagnostics into public health systems.

Uruguay’s progress toward implementing medical genomics reflects many of the structural limitations documented in other countries in the region. Although Uruguay is classified by the World Bank as a high-income economy [37], this designation does not accurately reflect the country’s level of investment in science or its actual readiness to implement genomic medicine at scale, where it may instead be considered an “emerging” nation [34]. Economic classification alone does not capture the availability of clinical-grade sequencing platforms or the regulatory and reimbursement frameworks required for routine genomic diagnostics [38]. These challenges are further compounded by the fact that genetic and genomic studies are not included in Uruguay’s national health benefit package (Plan Integral de Atención en Salud, PIAS) [39]. and therefore are not covered by the public or private healthcare systems. As a result, genomic testing is largely unavailable to the general population, with access depending on research initiatives, philanthropic support, or out-of-pocket payment, reinforcing existing inequities in the diagnosis and management of RDs.

In addition to limiting access to sequencing, these structural constraints also affect the ability to interpret genomic findings. Accurate genomic variant interpretation in the Latin American population is hampered by underrepresentation of these populations in global genomic databases [40]. This has a direct consequence in the diagnostic yield of RDs in such ethnicities. Accurate interpretation frequently depends on filtering out non-disease variants, which can be challenging if not all variants have been identified [41]. This issue is particularly relevant in admixed populations, where allele frequencies can vary substantially from those observed in more commonly studied groups. It is expected that incorporating population-specific allele frequencies derived from Uruguayan individuals can improve variant interpretation by reducing false positives. Variants that appear rare in external databases but are frequent in our local cohort may be deprioritized in diagnostic workflows, increasing specificity and avoiding overinterpretation.

We have developed a fully integrated workflow connecting sequencing capacity, bioinformatics processing, variant interpretation, and clinical genetics, fully relying on the development of human resources in both clinical and molecular genetics and bioinformatics. This structure was built from the ground up through collaboration between academic institutions and healthcare providers, as no prior organizational model or operational infrastructure existed within the health system. We established sequencing pipelines, implemented bioinformatics workflows, created multidisciplinary variant review teams, and developed standardized clinical reporting procedures. Together, these components enabled the first functional pipeline for RD genomic diagnostics in Uruguay.

In this work, we present the development and implementation of Uruguay’s national genomic medicine initiative for RDs, outlining the integrated framework built over the past decade and highlighting its transformative diagnostic impact on the healthcare system. We have sequenced 201 WES, 29 WGS and 11 mtDNA in RD patients in the context of the first medical genomics program in Uruguay. Our general diagnostic rate yielded 48% for exome and 31% for genome.

## Material and methods

### Sampling process

All participants were given written informed consent prior to enrollment. The first 29 patients of the program were assessed via Whole Genome Sequencing (WGS) at 30X within the framework of the Urugenomes project, approved by the Ethics Committee of the Institut Pasteur de Montevideo (URUGENOMES Project IP011-17/CEI/LC/MB). Subsequent participants were evaluated by Whole Exome Sequencing (WES) at 100X in the context of an extension of the URUGENOMES project. Later an addendum was issued by the ethics committee for the use of WES instead of WGS. Additionally, two previously unsolved cases were selected for pilot Long read Sequencing (LR-WGS) to explore its potential diagnostic contribution in individuals without molecular findings after prior WES/WGS analyses.

The majority of participants (94%) are pediatric patients recruited by the Department of Genetics of the Faculty of Medicine, Universidad de la República (UDELAR). Most of them have medical coverage by the public health system. Some pediatric patients have some other form of health coverage, and a few adult patients from the public health system were also included.

Since this program is strongly associated with a clinical genetic service, inclusion criteria were wide and oriented to those patients (or families) in whom a genetic disease was highly suspected, but no specific diagnosis was achieved after extensive clinical and other genomic evaluation. Hence a highly heterogeneous group of patients was analysed and in highly variable clinical scenarios. In addition, cases where a highly heterogeneous genetic disease was suspected (for instance: suspected genetic forms of epilepsy, syndromic developmental delay and RASopathies and their differential diagnosis) were also included. In some cases a clinical diagnosis was suspected or even confirmed on clinical grounds, although not a molecular specific diagnosis. In most cases no clear diagnosis was proposed previously.

### DNA extraction and sequencing strategies

Genomic DNA for WGS and mitochondrial DNA (mtDNA) analysis was extracted from 100 μL of whole blood using the QIamp® DNA Blood Mini kit (Qiagen, Germany) following the manufacturer’s instructions. Quality control was performed prior to sequencing; metrics included volume, concentration, and purity (for WGS, minimum conditions were 1 µg, 50 ng/µl, and an optical density 260/280 ratio between 1.8 and 2.0). WGS was performed at Macrogen (Seoul, South Korea) on an Illumina HiSeq X Ten platform using 150 bp paired-end reads, targeting an average depth of 30X. mtDNA was sequenced within our sequencing facility, on an Illumina MiSeq using two PCR-amplified fragments as previously described in Spangenberg et al. [42].

For all WES, wDNA was extracted from 200 µL of whole blood using the Quick-DNA™ Miniprep Plus Kit (Zymo Research, USA). Quality control was performed prior to sequencing; metrics included volume, concentration, and purity (minimum conditions were 3 µg, 50ng/µl, optical density 260/280 ratio between 1.8 and 2.0). WES was performed primarily at Macrogen on an Illumina NovaSeq platform using 150 bp paired-end reads, achieving an average depth of 100–150×. Additionally, a subset of 48 samples were sequenced at 3billion Inc. as part of a specific collaboration grant.

LR-WGS was performed using the PacBio Revio platform, generating HiFi (High-Fidelity) circular consensus reads at ~10X coverage (~90 Gb both samples). Library preparation and sequencing QC were carried out by Novogene following PacBio® HiFi sequencing standards. Quality metrics included volume, concentration, and purity (minimum conditions were 10 µg for the standard protocol, 50ng/µl, optical density 260/280 ratio between 1.8 and 2.0), as well as high molecular weight (HMW) integrity, verified by pulsed-field gel electrophoresis.

In this genomics program we have done NGS of the index case and no trios. If a candidate variant was obtained segregation analysis was done using Sanger sequencing.

### Bioinformatic analyses

Following sequencing, FASTQ files were securely transferred to encrypted, access-controlled local servers to ensure compliance with data protection standards for human genomic information. All subsequent bioinformatic analyses were performed in-house using a pipeline developed from the ground up for this program. This workflow integrates some locally implemented procedures with widely adopted external tools and adheres to internationally recognized best-practice guidelines, ensuring robust and reproducible variant calling and interpretation. The bioinformatic workflow includes the identification of single nucleotide variants (SNVs), insertions and deletions (INDELs), and different types of structural variants (SVs).

Raw sequencing data were initially assessed with FastQC [43]. High-quality reads were then mapped to the human reference genome (GRCh37) using the Burrows-Wheeler Aligner (BWA) [44]. Only uniquely mapped reads in proper pairs were retained for downstream analysis. Duplicate reads were marked and sorted.

Small-variant calling (SNVs and INDELs) was performed following the Best Practices recommended by the Genome Analysis Toolkit (GATK) [45]. Resulting variants were annotated using ANNOVAR [46], to integrate functional, genomic, and frequency-based information.

SVs were detected using Manta for both WGS and WES analysis (applying the *–exome* mode when appropriate), recognizing that SV detection in exome data has inherent sensitivity limitations [47]. Resulting SV calls were annotated with AnnotSV [48], integrating genomic context, regulatory annotations, and clinical evidence.

For patients requiring mitochondrial genome analysis, we additionally employed MToolBox [49] to characterize mitochondrial variants, haplogroup assignment, and heteroplasmy levels.

For LR-WGS analysis, PacBio HiFi reads were mapped to the GRCh37 reference genome using pbmm2 [50, 51], the aligner optimized for HiFi data. SVs were identified with pbsv [52] enabling sensitive detection of large INDELs, inversions, and other complex rearrangements. Resulting SV calls were annotated with AnnotSV, following the same annotation strategy used for long and short-read SVs [48].

Depending on the variant type, additional downstream analyses were performed as needed. For example, candidate frameshift variants underwent pathogenicity prediction using SIFT-Indel [53].

To evaluate the contribution of population-specific genetic variation to variant interpretation, we generated allele frequency data from our local cohort. To obtain cohort-specific allele frequencies, sequencing data from individual samples were processed to jointly call variants using GATK, followed by allele frequency estimation across the dataset using PLINK [54]. These cohort-derived frequencies provide a more accurate representation of the genetic variation present in the Uruguayan population and were incorporated into downstream filtering to reduce false positives and improve specificity in variant interpretation.

In some cases, the variant appeared to be of minor effect, obscuring the interpretation of its functional relevance and impact on the patient. However, after multiple checks it was often the only finding about the patient as compared to family settings, e.g., a subtle mitochondrial variant at high heteroplasmy levels. In those cases, we carefully analyzed the three-dimensional structure of the encoded protein, using homology modeling [55] and then AlphaFold2 after the code was released [56]. We succeeded in providing molecular mechanistic explanations to clinicians and patients, for the biochemistry underlying the phenotype, and qualitative functional extrapolations for cells and tissues [42, 57].

All statistical analyses and downstream data handling and variant filtration were performed in R [58]. Putative clinically relevant variants were visually inspected using the Integrative Genomics Viewer (IGV) [59, 60] to assess read support, mapping quality, strand balance, and potential sequencing artifacts [61].

### Variant classification and clinical interpretation algorithms

**Fig. 1:**
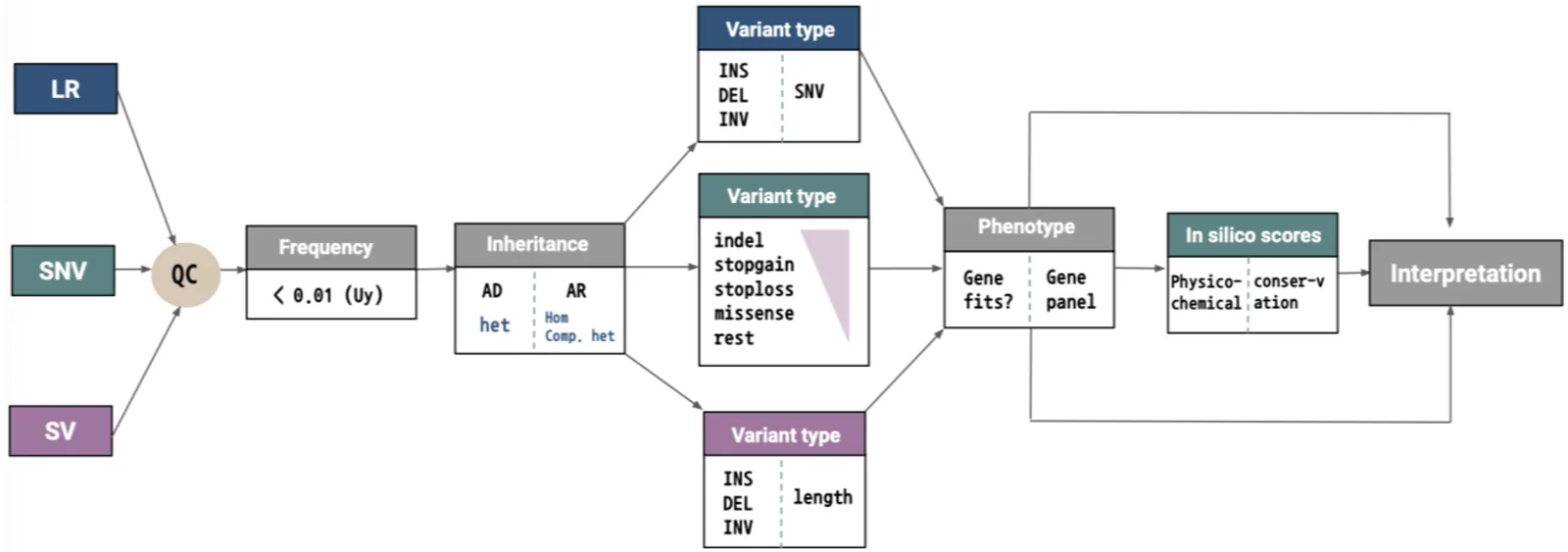
Variant filtering and prioritization workflows depending on sequencing strategy. Variants detected by WES, WGS, or LR-WGS (including SNVs, INDELs, and SVs) follow a unified filtering process consisting of quality control, population frequency thresholds based on the Uruguayan cohort, inheritance modeling, variant consequence categorization, phenotype-gene matching (including optional in silico gene panels), and in silico pathogenicity scoring. High-priority candidates identified through this workflow proceed to the clinical interpretation stage

Variant interpretation follows a standardized, stepwise filtering and prioritization workflow that integrates variant type, population frequency, inheritance mode, phenotypic relevance and in silico evidence of pathogenicity. The main steps of this process are summarized in Fig. 1. Although this sequential prioritization was applied consistently across WES, WGS, and LR-WGS, specific filtering paths varied depending on the type of variants detectable by each modality.

For nuclear variants, initial filtering was based on population frequency thresholds (<1% for recessive models and <0.5% for dominant models), incorporating both global databases (including gnomAD v2.1.1, ExAC, and 1000 Genomes Project) and cohort-specific allele frequencies derived from the Uruguayan population [41, 62, 63]. Variants were then subsequently evaluated under the inheritance model consistent with the clinical presentation: hemizygous for X-linked recessive phenotypes, homozygous or compound heterozygous for autosomal recessive phenotypes, and heterozygous for autosomal dominant presentations or X-linked *loci* in females.

SNVs and INDELs were then prioritized by functional impact (e.g., missense, truncating, canonical splice-site, predicted splicing effects). SVs detected from WGS and LR-WGS were incorporated into the same prioritization workflow after frequency and inheritance filtering. Across all sequencing strategies, candidate variants were manually evaluated for gene-phenotype concordance using curated disease-gene resources (OMIM) and available literature [64]. Phenotypic information, including Human Phenotype Ontology (HPO) terms when available, was used to guide variant prioritization through expert clinical evaluation [65]. HPO terms were also used for estimating diagnostic yield for some phenotypes (for instance, Global developmental delay and Intellectual disability).

The analysis was always initially WGS or WES based. When appropriate, phenotype-driven in silico gene panels were applied to restrict prioritization to genes with a known or suspected relevance to the patient’s clinical presentation (mostly to simplify the manual curation of variants). These panels were primarily derived from publicly available or external resources, as well as from curated gene lists reported in the literature or databases like OMIM, and were adapted or complemented by expert-defined gene lists based on clinical evaluation.

In addition, ACMG Secondary Findings analysis was offered to all participants.Secondary Findings analysis was performed in patients who provided explicit informed consent to receive this information, following current ACMG recommendations. In those, variants mapping to the 73–86 ACMG-SF medically actionable genes (depending on the version) were systematically reported [66–70]. For statistical analysis (see Results) the genes in the list were analysed in all cases, but not reported in the few cases that opted out.

WGS incorporated an additional evaluation layer, enabling prioritization of rare noncoding variants (regulatory, promoter, and deep intronic) when previously reported or supported by biological plausibility.

For patients undergoing mitochondrial genome sequencing, interpretation followed mtDNA-specific criteria: variants with heteroplasmy ≥ 10%, located in coding regions or rRNA/tRNA genes, were retained for evaluation, while variants defining haplogroups or located in the D-loop were excluded. Prioritization was based on functional impact, conservation, and concordance with known mitochondrial disease phenotypes.

Across all sequencing modalities, candidate variants were systematically assessed using annotation-derived in silico pathogenicity predictors, including widely used tools such as SIFT, PolyPhen-2, MutationTaster, PROVEAN, and ensemble scores such as REVEL, CADD, and MetaSVM/MetaLR [71–77]. These predictors were applied as implemented in the annotation framework and according to best practices at the time of analysis. Conservation metrics (e.g., GERP++, phyloP, phastCons) were also considered [78–80]. Splicing effects were evaluated based on variant annotation (canonical splice-site variants) and, when available, supported by dedicated prediction tools (e.g., SpliceAI) [81]. Indel variants were further assessed using tools such as SIFT-Indel when applicable [53].

Variant classification was performed through a semi-manual process combining automated annotation and filtering with expert clinical review. Annotated variants were provided as a prioritized list (based on population frequency and predicted effect) and evaluated by clinical geneticists. Variants were reviewed by clinical geneticists based on first-hand knowledge of the clinical presentation and family history, selecting the ones deemed of interest primarily based on phenotype match. Final pathogenicity classification followed ACMG/AMP guidelines [82], incorporating published refinements when applicable [83] External tools were used as supportive resources for the interpretation of specific variants, but no single automated platform was systematically applied. All shortlisted variants were visually validated in IGV to confirm read-level support, rule out artifacts, and assess alignment quality [59]. ClinVar was systematically used to assess previously reported variants and their clinical significance [84].

Familial segregation analysis (via Sanger sequencing) was performed in two situations: i. when this information was useful to classify the variant(s) (i.e.to check de novo or trans status, or to confirm the presence of the variant in affected relatives); ii. when presymptomatic relatives were available and would benefit from analysis (i.e. in actionable genes or symptomatic patients).

Diagnosis in each case was considered as definitive (pathogenic variants in heterozygous state for dominant disorders, pathogenic variants in hemizygous, homozygous or compound heterozygous state for recessive disorders or a pathogenic variant in combination with a likely pathogenic variant in compound heterozygous state for recessive disorders), *likely* (likely pathogenic variants in heterozygous state for dominant disorders, likely pathogenic variants in hemizygous, homozygous or compound heterozygous state for recessive disorders or a pathogenic variant in combination with a variant of unknown significance in compound heterozygous state for recessive disorders), *possible* (variant of unknown significance in heterozygous state for dominant disorders, a variant of unknown significance in homozygous state for recessive disorders or two variants of unknown significance in compound heterozygous state for recessive disorders, or pathogenic or likely pathogenic variant in heterozygous state for a recessive disorder (partial diagnosis)), and no diagnosis, based on [85]. Since we did proband singleton analysis, we did not find novel candidate genes which would be considered as a possible diagnosis until further data is gathered. Another category included for selected cases was *risk factor*, which includes the identification of variants that do not cause the primary phenotype but may increase susceptibility, modify severity, or contribute to a broader clinical picture. These include carrier findings for recessive conditions, variants of small effect size, or alleles associated with increased risk rather than monogenic causation.

## Results

### Implementation of a state of the art diagnostic and formative pipeline for rare disease in Uruguay

**Fig. 2:**
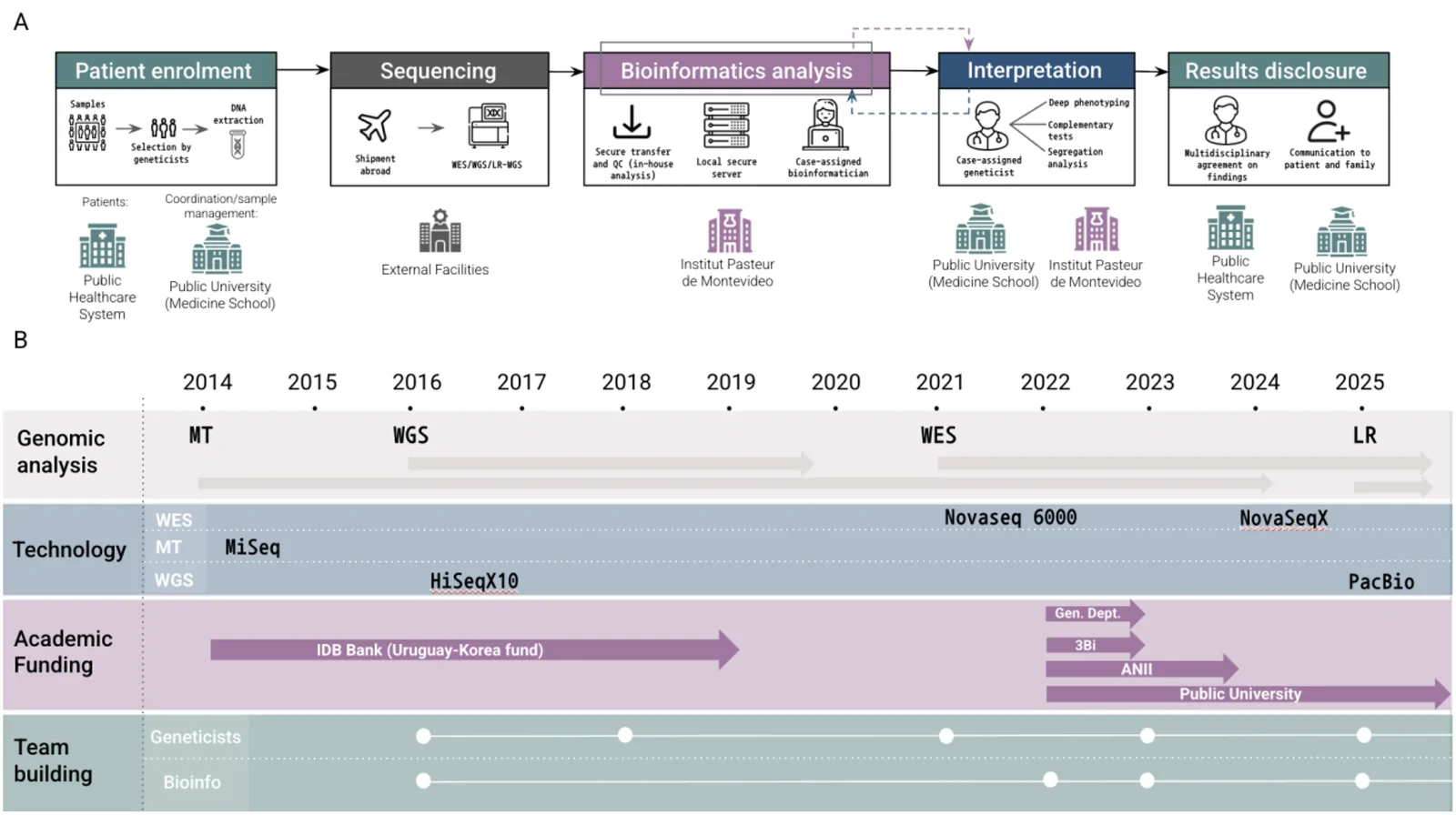
Clinical implementation of an inter-institutional genomic medicine workflow for rare diseases in Uruguay. **A**. Overview of the end-to-end workflow, including patient clinical evaluation and enrollment within the public healthcare system, sequencing performed at external facilities, in-house bioinformatics analysis, multidisciplinary clinical interpretation and coordinated results disclosure to patients and families. The workflow is based on an integrated academic–clinical collaboration involving the public healthcare system, the Faculty of Medicine of the Universidad de la República, and the Institut Pasteur de Montevideo. **B**. Timeline of the development and consolidation of genomic medicine capabilities in Uruguay (2014–2025), illustrating the progressive transition from mitochondrial DNA sequencing (MT), followed by whole-genome sequencing (WGS), the subsequent incorporation of whole-exome sequencing (WES), and the recent adoption of long-read sequencing (LR-WGS). Sequencing technologies include Illumina MiSeq, HiSeq X Ten, NovaSeq 6000, and PacBio platforms. The timeline also highlights the role of national and international academic funding initiatives and the parallel growth of specialized teams in clinical genetics and bioinformatics

An overview of the implementation of the rare disease medical genomics program including patient enrollment, sequencing, bioinformatics analysis, clinical interpretation and coordinated results disclosure to patients and families is shown in Fig. 2A. This is an academic program sustained by research funding, with core support from the Faculty of Medicine of the Universidad de la República and the Institut Pasteur de Montevideo.

The program was initially launched within the framework of a binational IDB Bank agreement between Uruguay and Korea (Fig. 2B, funding panel). After this initial phase, additional academic grants were secured to sustain its continuity and expansion. The COVID-19 pandemic years were particularly challenging for securing research funding. The program remains fundamentally an academic initiative whose long-term viability depends on the competitive research projects obtained by the participating teams. Given that research funding in Latin America is limited and highly competitive, the long-term stability and impact of the program may be compromised, underscoring the need for sustained investment to consolidate and expand its impact on the society.

Since its inception in early 2015, our genomics program has made significant strides in advancing education and professional development within the field of genomics in Latin America. Figure 2B (team building panel) shows formation instances such as intensive courses lasting from one to three weeks bringing worldwide human genomics experts to teach within the country, for example from the US, Europe and Asia. We also expanded the knowledge to the region given courses in other countries, eg. Brazil and Bolivia. Also, experts from Europe (representatives from solveRD program) were invited to give talks and workshops. Some of the courses were oriented for medical doctors, specifically geneticists, others for bioinformaticians and molecular biologists (Fig. 2B, team building panel). Several students of clinical genetics and other medical specialties used the patient data described here as part of their training (since 2021). In some cases, these were published as case reports and will be used as material for monographs and final degree projects [57, 86–88]. Hence, over these past years, we have trained approximately 20 researchers in Uruguay and several denizens in the region, equipping them with expertise in cutting-edge genomics techniques and analytical methodologies. In addition to human resource development, the program has fostered innovation through the establishment of start-up companies, contributing to the growth of a genomic technology ecosystem within our country.

The sequencing technology used within the first years was mitochondrial DNA at high coverage, done in house at the MiSeq. Shortly after, we were one of the firsts in the use of whole genome sequencing strategy. Furthermore, we switch to WES as it is cost effective and more sustainable given limited funding (Fig. 2B, genomics analysis). Sequencing was performed using Illumina platforms, and the specific instruments used are shown in Fig. 2B (technology panel).

### Description of samples included in the study with their corresponding phenotype

**Fig. 3:**
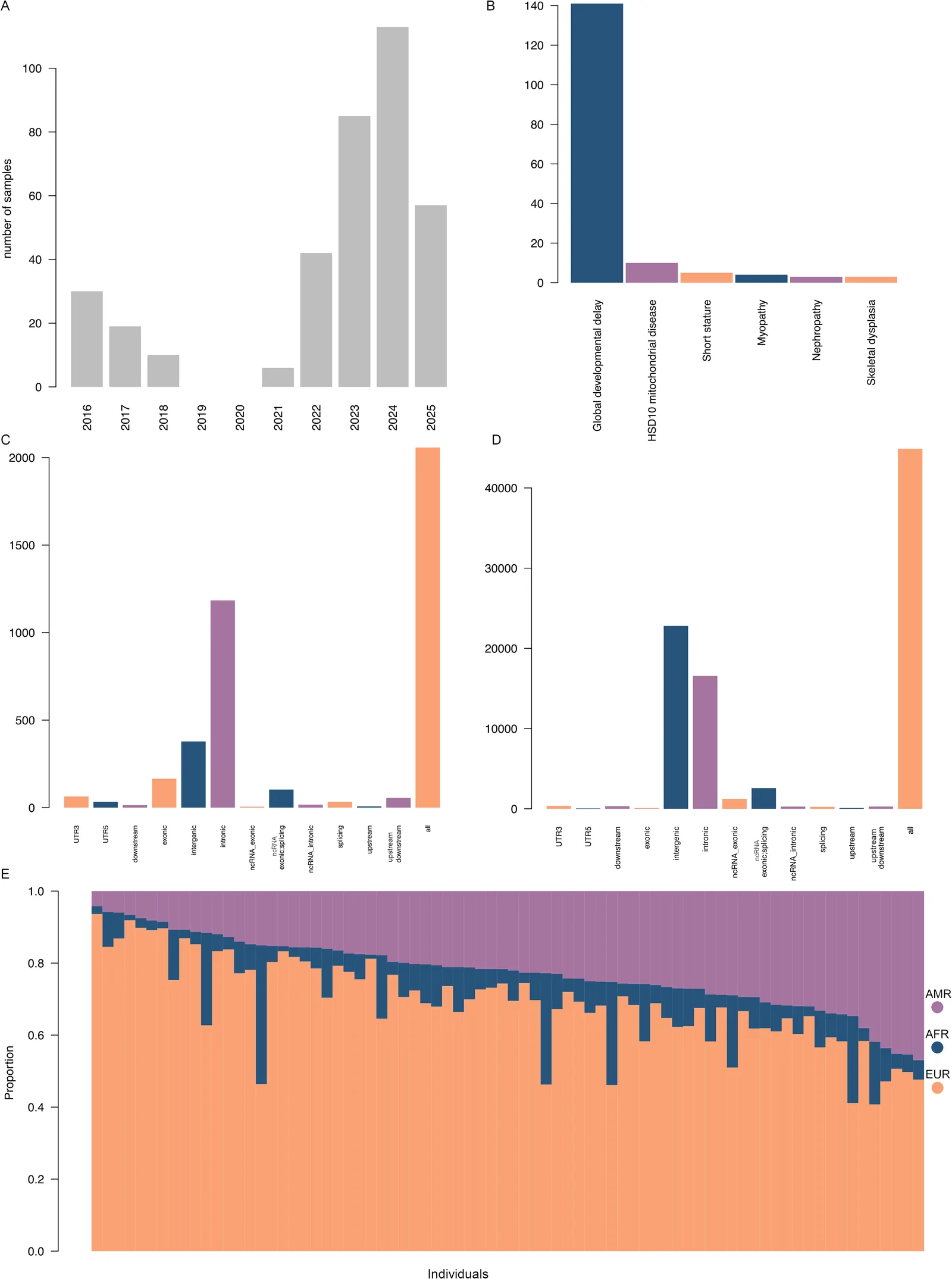
Cohort composition, sequencing activity, and population structure of the Uruguayan genomic dataset. **A**. Bar plot showing the number of samples sequenced per year between 2016 and 2025. **B**. Bar plot showing the distribution of most frequent primary Human phenotype ontology (HPO) terms assigned to the participants, summarizing the main phenotypic categories represented in the cohort. **C**. Bar plot showing the average of newly identified variants per exome. Variants are grouped by functional annotation category: exonic, intronic, splicing, UTR3, UTR5, upstream, downstream, and non-coding RNA (ncRNA) regions. **D**. Bar plot showing the average of newly identified variants per genome. **E**. Global ancestry distribution inferred for a representative sample (*n* = 76) of the patients included. Orange represents European ancestry, violet Amerindian ancestry and blue African ancestry

The study cohort comprises patients referred to the program over multiple years, reflecting the progressive consolidation of the diagnostic workflow. The number of samples analyzed per year varied over the course of the program (Fig. 3A). Following an initial phase of implementation, sequencing activity temporarily decreased between 2016 and 2019. This reduction reflects the use of a single, time-limited funding source that was primarily allocated during the first stages of the program (as shown in the academic funding timeline in Fig. 2A). During this period, efforts were also redirected toward in-depth analysis of the initially generated data, establishment of the bioinformatic pipeline and training of human resources. This phase was followed by a period impacted by the COVID-19 pandemic, which constrained sequencing capacity and redirected institutional efforts and priorities. In subsequent years, the number of patients sequenced increased steadily, coinciding with expanded funding through national scientific projects, consolidation of the bioinformatic and clinical workflow, and the progressive integration of the programme into routine diagnostic practice of the main clinical genetics service in the country. The apparent reduction in 2026 reflects a partial year of data collection.

Overall, 44% of the patients enrolled in the program were female. The phenotypic spectrum of the cohort is summarized in Fig. 3B. Global developmental delay (HP:0001263) was the most frequent primary phenotype, accounting for 70% of cases. The second most frequent category, representing approximately 5% of cases, is HSD10 mitochondrial disease (MONDO:0010327) and short stature (HP:0004322). HSD10 mitochondrial disease does not correspond to a single HPO term, but rather to a clinical diagnosis encompassing multiple HPO features within the broader category of mitochondrial disorders. The third term is short stature (HP:0004322) accounting for 2% of the cases. Consistently, the most frequent secondary associated HPO term across the cohort was abnormal facial shape (HP:0001999) (Figure S1).

The majority of enrolled individuals were pediatric patients, with only 13 adults included in the cohort. Almost all patients were referred from the public healthcare system. Prior to enrollment, 140 patients had undergone conventional karyotype analysis and 29 had received chromosomal microarray testing. Consanguinity was reported in 13 families, consistent with previously described low consanguinity rates in the Uruguayan population [89]. Regarding sequencing strategies, 29 of the 203 patients underwent whole-genome sequencing (WGS), 172 were analyzed by whole-exome sequencing (WES), of whom nine also received targeted mitochondrial DNA (mtDNA) analysis, while two patients were evaluated exclusively using mtDNA sequencing.

We characterized the landscape of genetic variation identified through exome and genome sequencing in the Uruguayan cohort. Figure 3C and D summarize the average number of newly identified variants per individual detected by WES (~2057) and WGS (~44887), respectively, stratified by functional annotation category. Across both sequencing strategies, a substantial number of previously unreported variants was identified, underscoring the limited representation of the Uruguayan population in global reference databases and highlighting the importance of generating population-specific genomic data to improve variant interpretation. In WES (Fig. 3C), most newly identified variants were located within intronic (1184 on average per patient) and exonic (165 on average per patient) regions captured by the enrichment design, with additional contributions from splicing sites (32 on average), untranslated regions (3’UTRs 63 and 5’UTR 33 on average), and proximal upstream and downstream regions (7 and 14, respectively). In WGS (Fig. 3D), a markedly higher number of novel variants was observed across non-coding categories, including intronic (16564), intergenic (22793), upstream/downstream (285), and ncRNA-associated regions (1214 ncRNA exonic, 281 ncRNA intronic, 2582 ncRNA exonic;splicing), reflecting the comprehensive genomic coverage provided by WGS. Notably, the number of newly identified exonic and canonical splicing variants per individual was of similar magnitude to that observed in WES.

Additionally, we compared the total variant burden identified by WES and WGS across functional categories (Table 1). At the individual level, WES identified on average ~24500 exonic variants and ~3700 splicing variants per individual, whereas WGS yielded comparable values for coding regions, with ~24700 exonic and ~3300 splicing variants per genome. These small differences (<1%) between technologies indicate that the exome capture strategy does not substantially bias the detection of coding and splice-site variation, supporting the robustness of WES for interrogating protein-coding regions in a clinical diagnostic setting. Substantial relative increases of variants were observed in WGS vs WES, as expected, for intronic variants (1702808 vs 110,108, ~15-fold increase), intergenic (2584237 vs 44,776, ~58 fold increase), upstream variants (27311 vs 888, ~31-fold increase), downstream variants (32431 vs 1632, ~20-fold increase) and ncRNA-associated variants, including ncRNA intronic variants (278777 vs 2475, ~113-fold increase). These results highlight the complementary nature of WES and WGS: WES provides an efficient and focused strategy for detecting clinically relevant coding variation, whereas WGS expands variant discovery into non-coding genomic regions which account for the vast majority of newly identified variants. Additionally, we determined the number of coding variants across types of coding variants (frameshift, non-frameshift, stopgain, stoploss, synonymous and non-synonymous) reported in WES and WGS (Table S1). On average each individual has 226 frameshift (131 frameshift deletion and 95 insertion), 337 non-frameshift (196 deletion and 181 insertion), 11,257 non-synonymous, 11,603 synonymous, 108 stopgain and 21 stoploss variants (as detected by WGS). Very similar values were observed with WES (Table S1).

**Table 1:** Total variants identified by WES and WGS across functional annotation categories. For each category, values are reported as the average number of variants per individual and as the total number of variants aggregated across individuals analyzed (WES uses a representative subset of patients, *n* = 72; while for WGS all the patients were included, *n* = 29)

	WES		WGS	
	Average per individual	All individuals (n = 72)	Average per individual	All individuals (n = 29)
exonic	24459	4329261	24698	716231
UTR3	6138	1086441	44882	1301588
UTR5	3295	583235	7682	222787
splicing	3703	655613	3284	95227
downstream	1632	288926	32431	940493
intergenic	44776	7925332	2584237	74942882
intronic	110108	19489156	1702808	49381435
ncRNA_exonic	882	156095	16187	469434
ncRNA_exonic/splicing	9247	1636875	31231	905700
ncRNA_intronic	2475	438116	278777	8084534
upstream	888	157097	27311	792025

### Impact of population-specific data on variant detection and prioritization

To better describe the population we determined the number of ‘private’ variants detected in each exome. For this we included only variants with no previous description in dbSNP data base and not seen in Gnomad and ExAc databases (eg. frequency = 0). Figure 3Cshows the mean distribution of newly detected variants per exome, for a total of 2094 new variants per exome on average. Table 1 shows the average number of variants detected in each individual divided by type of variant. Additionally, we estimated the genomic ancestry composition for a representative sample (*n* = 76) of individuals (admixture plot in Fig. 3D), revealing average cohort proportions of 69.0% European, 22.6% Indigenous, and 8.4% African ancestry. This concurs with previous work describing ancestries in the country [90] and specifically in the Uruguayan public health system [91].

In this context, to assess the impact of population-specific allele frequencies on variant interpretation we estimated allele frequencies using only WES data from our cohort. Among variants initially classified as rare (minor allele frequency MAF < 0.1% in public population databases), 15.8% of homozygous variants and 20.5% of heterozygous variants were found to have a MAF exceeding 1% in our local cohort. These variants were deprioritized during diagnostic interpretation, as their high frequency suggests they are unlikely to be pathogenic when investigating rare disorders. This filtering step significantly reduced the number of candidate variants per sample, increasing the specificity of variant prioritization and illustrating the importance of population-specific reference data for accurate genomic interpretation in underrepresented populations.

### Next generation sequencing improves diagnostic yield of previously undiagnosed patients

For this work, we used the classification proposed by [85] to assess the diagnostic rate (see methods 2.4). Summary results of each classification is found in Table 2.

**Table 2:** General diagnostic outcome categories across the cohort, considering all the cases (ALL), the primary HPO term (HP:0001263) and stratification by sequencing strategy (WGS and WES). Diagnostic rate is calculated by considering categories “definitive” and “likely” as success events divided by the total

	ALL	HP:0001263	WGS	WES
Definitive	61	45	8	51
Likely	33	18	1	32
Possibile	49	37	7	42
Risk factor	1	1	0	1
No diagnosis	59	42	13	46
**Rate**	46%	44%	31%	48%

The overall diagnostic rate (considering all technologies and all phenotypes) is 46% when considering the category ‘definitely’ and ‘likely’ as diagnosis success. When considering only the cases with associated primary HPO term HP:0001263 (Global developmental delay) diagnostic rate is 45%. Considering only mitochondrial DNA patients (11) the diagnostic rate was also 45%.

In 24.4% of cases only Variants of Uncertain Significance (VUS) potentially associated with the phenotype were reported. In 6 (2,9%) additional cases an incomplete diagnosis (i.e., a likely pathogenic (LP) or pathogenic (P) variant in heterozygous state for a recessive condition) was made.Those were considered as “Possible” diagnoses.

In 5.4% of patients in whom the diagnosis was considered definitive or likely (*n* = 92), more than one genetic disease or a phenotype caused by more than one genetic disease (compound phenotype) was found. A detailed description of case-level variants, essential phenotypic descriptors (HPO), and age and sex of each case are in supplementary table S2.

### Reanalysis could contribute to increases in diagnosis

Reanalysis of sequencing data, both genome and exome, was performed in some selected cases, mainly at the request of patients or their treating physicians (and at least three years after the primary analysis). There were about 10 reanalyzed cases, so the results do not allow us to draw conclusions, but we did have cases in which reanalysis allowed us to identify possible additional causal variants. For example, in one case, a variant of uncertain significance was found in a gene (*RERE*) that could correspond to the phenotype, but one with a very nonspecific clinical presentation (Neurodevelopmental disorder with or without anomalies of the brain, eye, or heart). Since it was not possible to analyze the parents to see if it was *de novo* (it was heterozygous for a dominant disease), no progress could be made in its classification. In a reanalysis performed four years later, there were several submissions in ClinVar that classified it as likely pathogenic, so we considered it as a likely diagnosis.

### Long reads improve diagnosis in two full negative cases

For two samples that had WGS/WES done but no diagnosis obtained, we performed long reads sequencing using PacBio HiFi technology at 10X of depth of coverage.

The first case included a female patient presenting with severe global developmental delay (GDD), characterized by absence of expressive language, drug-resistant epilepsy, and subtle dysmorphic features. Brain MRI demonstrated complete agenesis of the corpus callosum, mild dilation of the lateral ventricles, and a focal indentation at the mid-portion of the right lateral ventricle. No additional structural abnormalities were identified. Given the combination of severe neurodevelopmental impairment, early-onset epilepsy, and characteristic neuroimaging findings, the patient was referred to our program for genomic evaluation. After the negative result of short reads WGS we analyzed the genome using long reads and obtained a total of 10,309 structural annotated variants. After frequency filtering we obtained 14 SVs, two of which were heterozygous deep intronic deletions (VUS) in the gene *PIGN* potentially related to the phenotype that need further evaluation, and that were previously not found in WGS.

The second case was a female child with bilateral congenital severe sensorineural hearing loss and early-onset retinal abnormalities. WES revealed an heterozygous likely-pathogenic variant in *SLC52A2* (autosomal recessive riboflavin transporter deficiency). After PacBio long read analysis we found 13,768 structural annotated variants. After frequency filtering we obtained five SV, a couple of which were candidate structural variants related to retinitis pigmentosa that could be explaining the diagnosis, but need further investigation (eg. RNA-seq, future work). No structural variants involving SLC52A2 were detected.

### Secondary, incidental and pre-reproductive findings

The vast majority of patients opted to receive secondary findings. It should be noted that throughout the study, different versions of the list of secondary findings recommended by the ACMG were used as it was updated. These were found in 5.9% of cases (*n* = 201, excluding two patients in whom only mtDNA was sequenced). In two cases affected parents were diagnosed retrospectively: in a case we detected a variant in *TGFBR2* gene; it was inherited from the father who suffered descending aortic aneurysm (Loeys-Dietz syndrome). In a case in whom no diagnosis was found, a secondary finding in *SDHB* gene was made; the variant was inherited from the mother who had a tumour removed from her neck many years ago; reviewing the Pathology report it was a paraganglioma (Pheochromocytoma/paraganglioma syndrome).

In some cases (1.5%, included in the 5,9% mentioned above), heterozygous variants for a recessive disease (e.g., Wilson’s disease) were found, so they were considered as potential risk for the individual and a variant of pre-reproductive interest.

In some cases, genes not included in this list were reported, but in the opinion of the medical team they warranted follow-up and preventive recommendations: for instance, cases of early-onset cataracts, glaucoma, or some forms of diabetes.

Most patients (83%) had findings that could be reported as reproductive risks (*n* = 165). Given the variability of the clinical situations we faced, not all of these cases were reported. The number of heterozygous variants for severe recessive diseases found ranged from zero to 9 in each individual. In cases where reportable variants were present, the average was 3 variants per individual (not considering those potentially related to the phenotype under study).

### Impact of genetic diagnosis on clinical management

For those cases considered as having a definitive or likely diagnosis we evaluated the clinical impact of having reached a diagnosis (*n* = 92). In 53% of cases, there is one or more potential impacts on the clinical management of these patients: specific treatments (1%), treatment guidelines (13%), risk and complication assessment (29%), cascade testing of other family members (25%), and genetic counseling for the patient and/or family members (52%).

## Discussion

Here we report the first implementation of a genomic medicine diagnostic program for rare diseases in Uruguay. The implementation of genomic technologies such as whole-exome sequencing (WES) and whole-genome sequencing (WGS) has transformed the diagnostic landscape of rare diseases worldwide. Today, their value in shortening the diagnostic odyssey is unquestionable: multiple international studies have demonstrated that early use of genomic sequencing significantly increases diagnostic yield, reduces unnecessary testing, and provides families with actionable information that can guide clinical care. Our experience aligns with this global evidence, reinforcing that WES/WGS should be considered an essential component of modern clinical practice for patients with suspected genetic disorders [57, 86–88, 92–96].

To date, we have analysed 203 patients using WES, WGS and mitochondrial DNA sequencing. The overall diagnostic yield of the program was 46% when considering cases classified as having “definitive” or “likely” diagnosis across all technologies and phenotypes. When stratified by sequencing modality, the diagnostic yield was 48% for WES and 31% for WGS. Among patients whose primary phenotype was global developmental delay (HP:0001263), the diagnostic rate was 44%; the mitochondrial DNA cohort had 36%. The lower diagnostic yield observed for WGS compared to WES in our cohort should be interpreted in the context of sample size and case selection rather than an intrinsic limitation in the technology. WGS was applied to a substantially smaller number of patients, and was used in the most challenging cases regarding diagnosis, including an unbiased and heterogeneous cohort of individuals with prior negative testing of complex phenotypes. Similar patterns have been reported in other cohorts, where diagnostic yield varies widely depending on cohort composition, prior genomic studies, availability of parental samples (e.g. for trio sequencing or variant assessment by Sanger), functional studies and interpretive resources rather than sequencing modality alone [14, 34]. Taken together, these findings place our results within the range reported by other genomic medicine initiatives, where diagnostic yields typically vary between ~28% and ~54% for similar approaches, but depending on study design and healthcare context [14, 30, 34, 35].

Beyond primary diagnostic yield, our results highlight additional dimensions of clinical value provided by genomic testing. In 5.4% of patients with a definitive or likely diagnosis, more than one genetic disease or a blended phenotype resulting from multiple pathogenic variants was identified. These findings illustrate the limitations of single-gene diagnostic paradigms and underscore the added value of genome-wide approaches in disentangling complex clinical presentations.

Incidental and secondary findings also represented a substantial component of clinical impact. Medically actionable secondary findings were identified in 5.9% of individuals who opted to receive this information, a proportion consistent with rates reported previously [97–99]. In some cases, secondary findings led to retrospective diagnoses in affected parents.

Evaluation of clinical impact among patients with definitive or likely diagnoses revealed that more than half (53%) experienced at least one change in clinical management, including tailored surveillance strategies, risk and complication assessment, cascade testing or relatives, and genetic counseling. Although direct therapeutic interventions were rare, these results are consistent with international experience showing that the principal clinical utility of genomic diagnosis in RD lies in prognosis clarification, preventive care, and long-term management rather than immediate treatment modification [100].

In addition to primary diagnostic performance, massive sequencing analyses can provide information of enormous clinical value: i. it allows for the reevaluation of phenotypes in which clinical diagnosis is not possible, is subject to error, or may be the result of a differential diagnosis not considered in the first instance; ii. It can explain cases of compound phenotypes (where the patient has more than one genetic disease or the phenotype results from the combined effect of several genetic variants); iii. it provides information on secondary findings of great preventive interest to the patient or their family in a significant percentage of cases; iv. it provides information of reproductive interest to the patient and possibly their family beyond the phenotype under study. Beyond the diagnostic outcome itself, the clinical impact of genomic testing is substantial. Identifying a molecular cause can clarify prognosis, inform surveillance strategies, enable access to targeted therapies when available, and provide accurate reproductive counseling. In several cases, genetic diagnosis also leads to changes in management or helps avoid interventions that are unlikely to be beneficial. As rare diseases often affect children and require long-term multidisciplinary care, the benefits extend not only to patients and families but also to the healthcare system through more efficient use of resources.

Given these advantages, we have proposed incorporating WES/WGS into the PIAS, Uruguay’s basic portfolio of health studies, as an urgent and strategic step. Doing so requires political commitment and the recognition that genomic diagnostics are not experimental tools but evidence-based clinical necessities [101, 102]. Access to these technologies should not depend on research grants or external collaborations, as this perpetuates inequities in care and limits long-term program sustainability. Ensuring public coverage would align Uruguay with international standards and guarantee that patients receive timely and equitable access to genomic medicine.

Maintaining and improving diagnostic yield will depend on the adoption of new state-of-the-art technologies, including long-read sequencing (LRS), RNA sequencing (RNA-seq), and integrative multi-omics approaches [12, 103–109]. Long-read sequencing is particularly powerful for detecting structural variants, repeat expansions, and to analyse complex genomic regions that are poorly resolved by short reads; our LRS pilot detected additional candidate SVs which needed further validation. In this context, RNA-seq could provide functional insights that help interpret variants of uncertain significance, uncover splicing defects, and reveal gene-expression signatures associated with disease [110–114].

Also LRS sequencing using Nanopore technology could give information on methylation status of regions of interest [115]. Incorporating these tools is essential not only for diagnosis but also for advancing our understanding of disease mechanisms, which in turn supports research toward novel therapies.

It has been demonstrated that reanalysis of data several years after the primary analysis improves diagnostic performance, especially based on the description of new associations between genes and diseases [116]. In our case, few patients underwent reanalysis, and the results are anecdotal. However, we had some cases in which reanalysis revealed candidate variants that had not been found previously.

Finally, the characterization of local populations is a critical component of rare disease genomics. Allele frequencies, population structure, and ancestry-specific variant backgrounds directly influence variant interpretation. Without population-specific genomic references, pathogenicity assessments risk being inaccurate, potentially leading to misdiagnosis or missed diagnoses. Developing genomic resources tailored to the Uruguayan population, including diverse and underrepresented groups, strengthens clinical interpretation, improves diagnostic confidence, and contributes to global efforts to expand diversity in human genomics.

In a region where research funding is limited and health-system resources are constrained, the success of this program highlights the importance of sustained investment, strategic planning, and strong academic-clinical partnerships. Continued support will be key to ensuring that patients with rare diseases in Uruguay receive accurate, timely, and comprehensive genomic diagnostics.

## Conclusion

Uruguay’s medical genomics program represents a significant step forward in rare disease diagnosis, addressing critical gaps in variant interpretation for underrepresented populations. By integrating NGS technologies and bioinformatics, this initiative has shortened the diagnostic odyssey for many patients, paving the way for precision medicine in the region. Beyond individual diagnoses this program demonstrates the feasibility and clinical utility of implementing genomic medicine within a public healthcare setting in Latin America. A key component of this process has been the sustained development of human resources, collaboration and team building. This emphasis on local capacity building was essential for transitioning genomic analyses from externally dependent research efforts to an integrated, reproducible, and clinically actionable diagnostic pipeline. Moving forward, expanding public coverage, strengthening national reference datasets, and incorporating emerging technologies will be essential to further improve diagnostic yield and equity, and to ensure the long-term sustainability of genomic medicine as part of routine clinical practice.

## Electronic supplementary material

Below is the link to the electronic supplementary material.

Supplementary material 1

Supplementary material 2

Supplementary material 3

Supplementary material 4

## Data Availability

The Uruguayan genomic data supporting the findings of this study are not publicly available due to privacy and ethical restrictions. Aggregated, population-level data can be shared upon request. Access to individual-level patient data requires approval from the Ethics Committee of the Institut Pasteur de Montevideo, as well as informed consent from the parents or legal guardians.
