# Supplementary material for: Implementation of a medical genomics program for rare diseases in Uruguay
Source: Orphanet J Rare Dis. 2026 Jun 24;21:319. doi: 10.1186/s13023-026-04387-2 (PMC13587377; doi:10.1186/s13023-026-04387-2)
Supplement: Supplementary file 1 [file 13023_2026_4387_MOESM1_ESM.pdf]

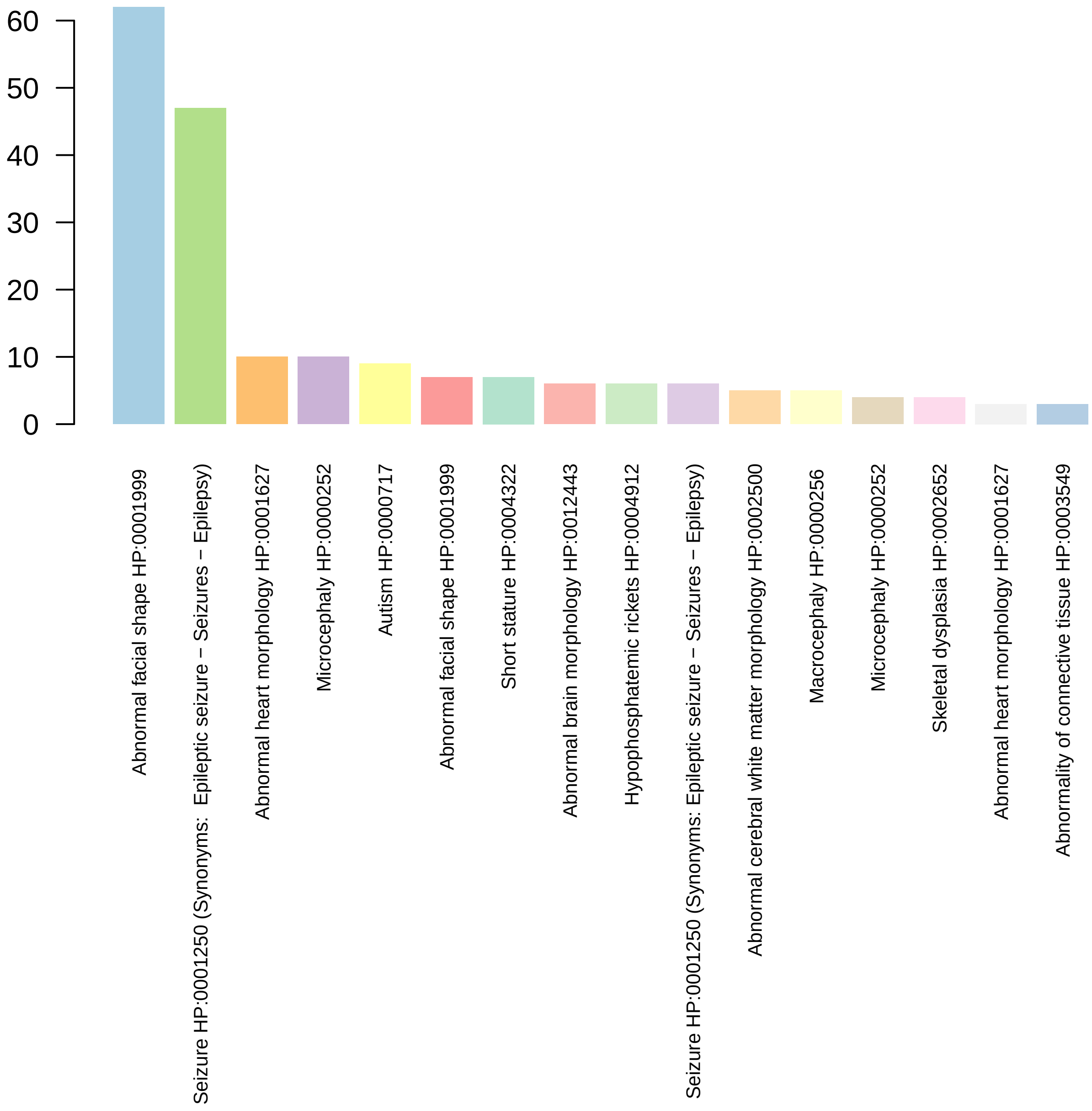

60
50
40
30
20
10
0
Abnormal facial shape HP:0001999
Seizure HP:0001250 (Synonyms: Epileptic seizure – Seizures – Epilepsy)
Abnormal heart morphology HP:0001627
Microcephaly HP:0000252
Autism HP:0000717
Abnormal facial shape HP:0001999
Short stature HP:0004322
Abnormal brain morphology HP:0012443
Hypophosphatemic rickets HP:0004912
Seizure HP:0001250 (Synonyms: Epileptic seizure – Seizures – Epilepsy)
Abnormal cerebral white matter morphology HP:0002500
Macrocephaly HP:0000256
Microcephaly HP:0000252
Skeletal dysplasia HP:0002652
Abnormal heart morphology HP:0001627
Abnormality of connective tissue HP:0003549
