# Supplementary material for: Implementation of a medical genomics program for rare diseases in Uruguay
Source: Orphanet J Rare Dis. 2026 Jun 24;21:319. doi: 10.1186/s13023-026-04387-2 (PMC13587377; doi:10.1186/s13023-026-04387-2)
Supplement: Supplementary file 2 [file 13023_2026_4387_MOESM4_ESM.docx]

**Legends supplementary material**

**Figure S1.** Frequency of secondary associated HPO term across the cohort.

**Table S1**. Number of coding variants across types of coding variants reported in WES and WGS.

**Supplementary Table 1.** Variant(s) found in individual cases considered as diagnosed (corresponding to the categories of “Likely” and “Definitive” (n = 92)), main phenotypic descriptors (HPO terms) and essential individual data.
